# Comparison of the Efficiency and Safety of Total Ankle Replacement and Ankle Arthrodesis in the Treatment of Osteoarthritis: An Updated Systematic Review and Meta‐analysis

**DOI:** 10.1111/os.12635

**Published:** 2020-03-29

**Authors:** Yuhan Li, Jinquan He, Yongcheng Hu

**Affiliations:** ^1^ Department of Biological Sciences Auburn University Auburn Alabama USA; ^2^ The First Department of Foot and Ankle Surgery Tianjin Hospital Tianjin China; ^3^ Department of Orthopaedic Oncology Tianjin Hospital Tianjin China

**Keywords:** AAD, Ankle Arthrodesis, Meta‐analysis, Osteoarthritis, TAR, Total Ankle Replacement

## Abstract

While osteoarthritis is a common degenerative disease, ankle osteoarthritis is a subdivision that has received little attention. Two effective ways to treat osteoarthritis of the ankle are total ankle replacement (TAR) and ankle arthrodesis (AAD). Whether TAR or AAD is more beneficial for treatment is controversial. The purpose of this meta‐analysis was to compare the efficiency (clinical outcome and patient satisfaction) and safety (complications and survival) of these two procedures. The Preferred Reporting Items for Systematic Reviews and Meta‐Analyses (PRISMA) statement was performed as a guideline for this study. Three electronic databases, PubMed, Web of Science, and Cochrane Library, were searched up to May 2019, with no language restrictions. Prospective or retrospective comparative studies were identified. The outcomes included clinical outcome, patient satisfaction, complications, and survival. Review Manager (Revman) 5.3 software was used to conduct the data analysis. We only selected literature from the past 5 years (no earlier than 2015). Seven comparative studies were included. There were six cohort studies and one cross‐sectional study. The Newcastle–Ottawa Scale (NOS) was used to assess the quality of cohort studies, and The Agency for Healthcare Research and Quality (AHRQ) checklist was chosen to assess the quality of cross‐sectional studies. No significant difference was observed for efficiency and safety. Clinical outcome was included in five studies with four different scoring systems. Two of them used the American Orthopaedic Foot & Ankle Society (AOFAS) questionnaire scores to assess the two procedures (mean difference, −4.26; 95% confidence interval [CI], −11.37–2.85; *P* = 0.24; *I* ^2^ = 1%). Patient satisfaction (risk ratio [RR], 0.96; 95% CI, 0.65–1.40; *P* = 0.82; *I* ^2^ = 54%), complications (RR, 1.15; 95% CI, 0.16–8.21; *P* = 0.89; *I* ^2^ = 84%), and survival (RR, 1.91; 95% CI, 0.33–11.08; *P* = 0.47; *I* ^2^ = 90%) showed no significant difference between the TAR group and the AAD group. This meta‐analysis showed no statistically significant difference between TAR and AAD in clinical outcome, patient satisfaction, complications, and survival. This revealed that TAR and AAD could appear to have similar results in these aspects. Therefore, the present results are not sufficient to conclude which of these two methods is better. Further studies are needed to obtain more clues.

## Introduction

Osteoarthritis (OA) is a degenerative disease that affects joints and their cartilage, leading to the loss of structure and function^1^, ^2^. Osteoarthritis affects several joints, such as the knee, the hip, and the ankle. Because the incidence of ankle osteoarthritis is relatively low compared to other types of osteoarthritis, there is less discussion on this topic^2^. Two practical and well‐established ways to treat osteoarthritis of the ankle are total ankle replacement (TAR) and ankle arthrodesis (AAD).

AAD, also referred to as ankle fusion, is a widely used treatment for ankle osteoarthritis. The use of this type of treatment can result in good patient satisfaction and can provide pain relief and improvement in function, thus helping patients return to normal daily life^3^. It is regarded as a standard treatment for ankle osteoarthritis. TAR is also a useful surgical treatment strategy as an alternative treatment to AAD^4^. However, early ankle replacement was not acceptable nor popular due to the high probability of complications^4^. In recent years, this technique has been improved to achieve better results, including reducing pain and improving functional outcomes^5^. With the continuous progress and improvement in ankle replacement surgery, it is receiving more and more recognition.

Despite TAR and AAD being efficient surgical treatments for osteoarthritis, they have certain shortcomings. Ankle fusion is a traditional and popular operative treatment. However, it can lead to persistent alterations in gait, and may even lead to the development of osteoarthritis in other joints, such as the subtalar joint, the talonavicular, and the midfoot^3^, ^5^. For ankle replacement, the results for short‐term and mid‐term survivorship and functional outcomes are promising; however, the long‐term effects require further research, and the operation is complicated and challenging for doctors^4^, ^5^.

Because of the controversy over whether TAR or AAD is more advantageous and progressive, a comparative study of these two methods is vital to clinical research. There have been few previous studies and meta‐analyses comparing TAR and AAD; however, some new literature has been published. Therefore, it is necessary to conduct an updated systematic review and meta‐analysis. In our meta‐analysis, we included studies published in the past 5 years (from 2015 to 2019) and assessed clinical outcomes, patient satisfaction, complications, and survival to explore the efficiency and safety of these two procedures and then to determine which approach is more effective for osteoarthritis.

## Methods

The Preferred Reporting Items for Systematic Reviews and Meta‐Analyses (PRISMA) statement was used to guide the study^6^.

### *Information Sources and Search Strategy*

The relevant works of literature selected in this study were mainly from three electronic databases up to May 2019, with no language restrictions: PubMed, Web of Science, and Cochrane Library. The keywords identified in this search were Medical Subject Headings (MeSH) “Osteoarthritis,” and all synonyms (free terms), MeSH “Arthroplasty, Replacement, Ankle” and all synonyms (free terms), MeSH “Arthrodesis,” “Arthrodeses,” “Ankle Arthrodesis,” and “Ankle fusion.” The combination of these MeSH terms and free terms was then applied.

### *Eligibility Criteria*

The following criteria for inclusion were applied: (i) population: adult patients with osteoarthritis; (ii) intervention: treatment with TAR; (iii) comparison: treatment with AAD; (iv) outcome: efficiency (clinical outcome and patient satisfaction) and safety (complications and survival); and (v) design: prospective or retrospective comparative studies (randomized controlled trials [RCT] and non‐randomized controlled studies [included observational studies]). The duplicated studies were excluded.

### *Data Collection*

The first author extracted all information about patients and treatments. These data involved: (i) the first author, year of publication, study type, number of subjects, mean age of subjects, and follow up’ (ii) outcome measures; and (iii) intervention characteristics of the TAR groups and the AAD groups. The other author reviewed and checked the extracted data. Any disagreements between the two authors were resolved by discussion.

### *Quality Assessment*

The Agency for Healthcare Research and Quality (AHRQ) checklist was chosen to assess the quality of cross‐sectional studies^7^. It included 11 quality items, and “yes,” “no,” and “unclear” could be applied to each item.

The Newcastle–Ottawa scale (NOS) was selected to assess the quality of other non‐randomized controlled studies^8^. There were three domains (selection, comparability, and outcome) and a total of eight detailed quality items in this scale. In “selection” and “outcome” domains, a maximum of one star could be awarded for each quality item. In the “comparability” domain, a maximum of two stars could be given. More stars obtained meant higher quality assessed.

For the included studies that were RCT, the Cochrane Collaboration's tool for assessing the risk of bias was appropriate^9^. The evaluation criteria were as follows: random sequence generation, allocation concealment, blinding of participants and personnel, blinding of outcome assessment, incomplete outcome data, selective reporting, and other bias. Because no RCT studies were included in this meta‐analysis, this quality assessment tool was not used.

Two authors conducted the quality assessment independently. Any disagreements between the two authors were resolved by discussion.

### *Data Analysis*

The statistical software used in this study for data analysis was Review Manager (Revman) 5.3. For the dichotomous outcomes, the selected effect size was the risk ratio (RR) with a 95% confidence interval (CI), and the chosen method was the Mantel–Haenszel method. Weighted mean difference (WMD) with 95% CI was selected for the continuous outcomes. The *I* ^2^‐statistic test was used to detect the statistical heterogeneity between studies. If the value of *I* ^2^ was less than 50%, indicating that the heterogeneity was low, the fixed‐effects model was chosen. Otherwise, the random‐effects model was used because of the high heterogeneity. A *P*‐value less than 0.05 was considered statistically significant. Due to the small number of studies included (fewer than 10), the publication bias was not assessable, and the Begg funnel plots were not used to indicate potential publication bias.

## Results

### *Study Selection*

**Figure 1:**
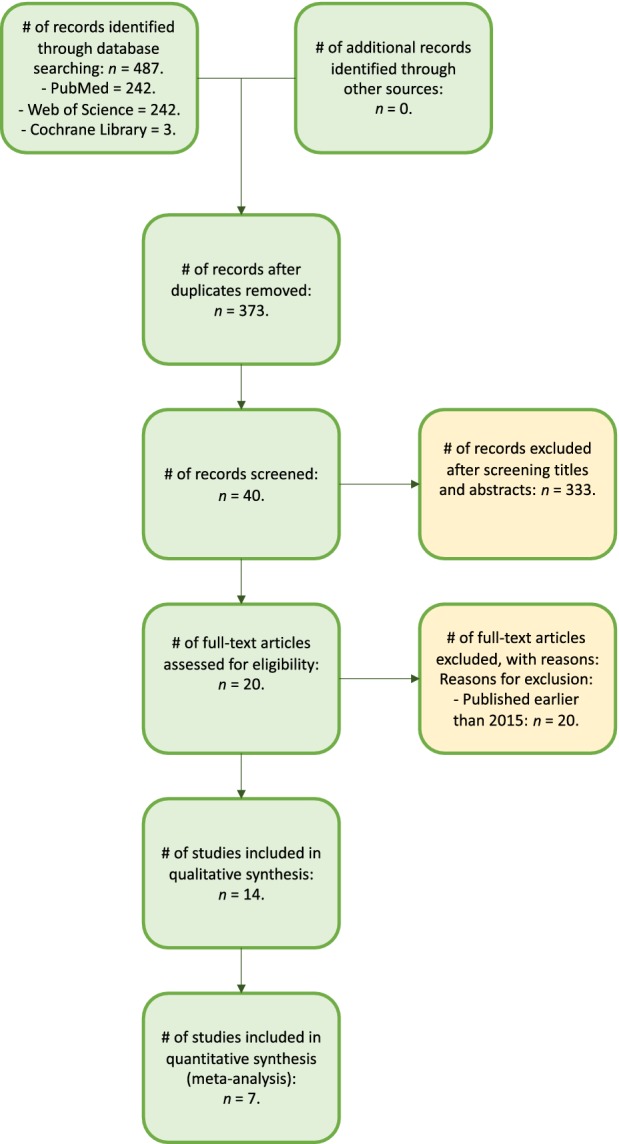
Study selection flow diagram (Preferred Reporting Items for Systematic Reviews and Meta‐Analyses [PRISMA]).

A total of 487 studies were identified and extracted from the initial three databases by using the inclusion criteria and the data collection strategy mentioned above. Among them, there were 242 works of literature from PubMed, 242 from Web of Science (only included trials), and 3 from Cochrane Library (only included trials). A total of 114 studies were excluded because of duplication and 333 studies were excluded after screening titles and abstracts because they did not study the two treatments (TAR and AAD) comprehensively or only studied one of them. The remaining 40 articles were reviewed by reading full texts to obtain more details. Of these, 20 were excluded because they were published earlier than 2015. There were 6 works of literature excluded because they did not clearly compare the efficiency and safety of the two therapies (TAR and AAD), and another 7 studies were excluded because they could not be studied in the quantitative synthesis. Finally, 7 studies^10^, ^11^, ^12^, ^13^, ^14^, ^15^, ^16^ were included in the meta‐analysis because they met the eligibility criteria. The process of the selection is shown in Fig. 1.

### *Study Characteristics*

Only studies from 2015 to 2019 (the past 5 years) were included. A total of 1280 patients were included in the 7 studies selected, of which 927 were treated with TAR and 353 with AAD. The follow‐up cycles were provided in all 7 studies, with the shortest one being 12.0 months and the longest being 77.0 months, while 5 studies^12^, ^13^, ^14^, ^15^, ^16^ showed the average age of patients. Five studies^10^, ^12^, ^13^, ^15^, ^16^ involved clinical outcome, 2 studies^12^, ^13^ presented patient satisfaction, two studies^13^, ^14^ compared complications, and four studies^11^, ^12^, ^13^, ^14^ reported survival. Because of the lack of directly given data, the standard deviations were estimated for the clinical outcome part of the studies by Mehdi and Pedowitz^13^, ^15^. Table 1 depicts the specific study characteristics and more details.

**Table 1:** Summary of characteristics of the studies included in the meta‐analysis

The First author	Year	Study type	Number of subjects	Mean age of subjects	Follow‐up (months)	Clinical outcome	Patient satisfaction	Complications	Survival
Chorpa^10^	2017	Retrospective	TAR:12 AAD:12	TAR:NR AAD:NR	TAR:56.4 AAD:56.4	FAAM	NR	NR	NR
Croft^11^	2017	Retrospective	TAR:362 AAD:169	TAR:NR AAD:NR	TAR:40.8 AAD:40.8	NR	NR	NR	TAR:62 AAD:8
Henricson^12^	2016	NR	TAR:7 AAD:7	TAR:61.1 AAD:61.1	TAR:77.0 AAD:66.4	SEFAS	TAR:7 AAD:6	NR	TAR:2 AAD:1
Mehdi^13^	2019	Retrospective	TAR:25 AAD:25	TAR:60.0 AAD:62.0	TAR:65.0 AAD:68.0	AOFAS; Pain VAS	TAR:16 AAD:20	TAR:7 AAD:2	TAR:9 AAD:1
Norvell^14^	2018	Prospective	TAR:395 AAD:98	TAR:63.3 AAD:54.2	TAR:12.0 AAD:12.0	NR	NR	TAR:33 AAD:17	TAR:16 AAD:13
Pedowitz^15^	2016	Retrospective	TAR:41 AAD:27	TAR:65.0 AAD:55.0	TAR:33.7 AAD:40.3	SF‐12MCS; SF‐12PCS; VAS; FAAM‐ADL; FAAM‐Sports	NR	NR	NR
Pinsker^16^	2015	Prospective	TAR:85 AAD:15	Total:61.2	TAR:30.0 AAD:30.0	SMFA; WOMAC; AOFAS; LEFS; FFI; AOS	NR	NR	NR

Survival = revision, re‐operation, or operation failure. ADL, activities of daily living; AOFAS, American Orthopaedic Foot & Ankle Society questionnaire; AOS, ankle osteoarthritis scale; FAAM, foot and ankle ability measure score; FFI, foot function index; LEFS, lower extremity functional scale; MCS, mental component summary; NR, not reported; PCS, physical component summary; SEFAS, self‐reported foot and ankle score; SF‐12, Short Form‐12; SMFA, short musculoskeletal function assessment; VAS, visual analogue scale; WOMAC, Western Ontario and McMaster Universities Osteoarthritis Index.

### *Quality Assessment*

The quality of the 7 studies was evaluated, with 6 cohort studies^10^, ^11^, ^12^, ^13^, ^14^, ^15^ and 1 cross‐sectional study^16^ included. Cohort studies were assessed using the NOS, and the cross‐sectional study was assessed using AHRQ checklist^7^, ^8^. The evaluation results and summary are shown in Tables 2 and 3.

**Table 2:** The Newcastle–Ottawa Scale (NOS) for assessing the quality of cohort studies

Study	Selection	Comparability	Outcome	Total scores
Chorpa (2017)^10^	***	**	*	6
Croft (2017)^11^	****	**	***	9
Henricson (2016)^12^	**	*	***	6
Mehdi (2019)^13^	***	**	**	7
Norvell (2018)^14^	****	**	**	8
Pedowitz (2016)^15^	***	**	**	7

**Table 3:** Agency for Healthcare Research and Quality (AHRQ) checklist for assessing the quality of cross‐sectional studies

Study	1	2	3	4	5	6	7	8	9	10	11
Pinsker (2015)^16^	+	+	+	U	U	U	−	+	−	+	U

Yes = +; no = −; unclear = U.

### *Data Analysis*

#### *Clinical Outcomes*

**Figure 2:**
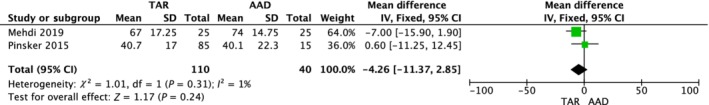
Forest plot for clinical outcome (American Orthopaedic Foot & Ankle Society [AOFAS] questionnaire scores) comparison between total ankle replacement (TAR) and ankle arthrodesis (AAD) groups.

Of 7 studies, 5 studies involved clinical outcomes, but they used various scoring systems. Two of them used the American Orthopaedic Foot & Ankle Society (AOFAS) questionnaire scores to assess the two groups: the TAR group and the AAD group (mean difference, −4.26; 95% CI, −11.37–2.85; *P* = 0.24; *I* ^2^ = 1%) (Fig. 2).

#### *Patient Satisfaction*

Two studies reported on patient satisfaction, involving 32 patients of the TAR group and 32 patients of the AAD group. Of these, 23 TAR patients were satisfied, and 26 AAD patients were satisfied.

**Figure 3:**
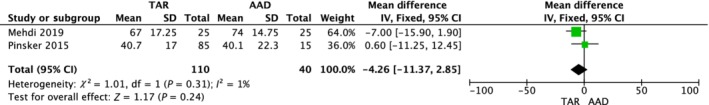
Forest plot for patient satisfaction comparison between total ankle replacement (TAR) and ankle arthrodesis (AAD) groups. [Correction added on 20 April 2020, after first online publication: figure 3 image has been corrected.]

The result showed that there was no statistically significant difference between the TAR group and the AAD group (RR, 0.96; 95% CI, 0.65–1.40; *P* = 0.82; *I* ^2^ = 54%) (Fig. 3).

#### *Complications*

**Figure 4:**
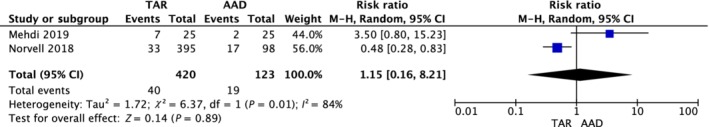
Forest plot for complication comparison between total ankle replacement (TAR) and ankle arthrodesis (AAD) groups.

Two studies involved complications, including 420 TAR subjects and 123 AAD subjects. The pooled data revealed that there was no statistical significance (RR, 1.15; 95% CI, 0.16–8.21; *P* = 0.89; *I* ^2^ = 84%) (Fig. 4).

#### *Survival*

**Figure 5:**
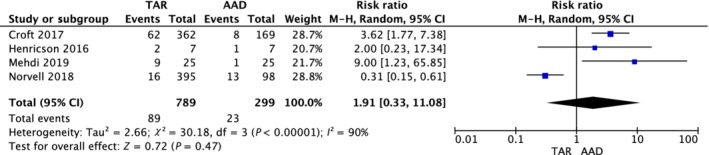
Forest plot for survival comparison between total ankle replacement (TAR) and ankle arthrodesis (AAD) groups.

Four studies presented survival details; that is, revision, re‐operation, or operation failure. Although there was no statistically significant difference found between the two groups, the RR showed that the risk of survival in the TAR group was relatively higher than that of the AAD group (RR, 1.91; 95% CI, 0.33–11.08; *P* = 0.47; *I* ^*2*^ = 90%) (Fig. 5).

## Discussion

This is an updated systematic review and meta‐analysis reporting on the comparison of the efficiency and safety of TAR and AAD in the treatment of osteoarthritis. This meta‐analysis was based on 7 studies published in the past 5 years (2015–2019).

Kim et al. conducted a similar meta‐analysis comparing TAR and AAD for end‐stage ankle arthritis in 2016^17^. The main finding of our study was relatively consistent with this previous meta‐analysis: TAR and AAD shared similar efficiency and safety in some aspects. Kim's work, like ours, concluded that TAR and AAD had no significant difference in clinical outcomes and patient satisfaction, but their conclusions showed that TAR patients had more re‐operations and complications than AAD^17^. In contrast, our results revealed no statistical difference between them. This discrepancy might occur because the works of literature included in our meta‐analysis were published more recently, and the surgical techniques might be improving. This might also be because the focuses on the details were different; for example, they were concerned about end‐stage ankle arthritis, while we were concerned about osteoarthritis.

Over time, TAR has made progress and has improved significantly in many areas over its predecessors^18^, ^19^, ^20^. Surgeons’ experience has also been increasing^19^. The same is true for AAD, where fusion rates have increased with the advent of new surgical techniques^21^. These factors can all lead to different results. This meta‐analysis only included studies published in the past 5 years because of the continuous progress of the procedures. Some early operations might be immature and showed poor results. If improvements in technology are not taken into account, the results may be biased. Therefore, we think it is necessary to select only recent studies for analysis. Besides, we did the screening of time periods after reading titles and abstracts, which could help not miss any high‐quality literature.

There are some potential limitations of this study. First, this meta‐analysis had a limited sample size. A small number of studies was included, and each study reported different outcome measures so that each outcome had a relatively small sample size. In addition, some of the included studies had few subjects. This might lead to potential bias or heterogeneity. For instance, various scoring systems were used to assess clinical outcomes in the included studies, and only two of the studies shared the same scoring system, AOFAS scores. For some rare complications, such a limited sample size might not be adequate. Besides, due to the different focuses of each included study, some assessments could not be performed because of the insufficient data. Therefore, further research is needed to resolve this issue.

Second, there was no RCT among the 7 studies included in this meta‐analysis, which were all observational studies. RCT is a relatively challenging type of research, so there are fewer RCT studies than other types of studies. Due to the characteristics of this study, ethical issues also need to be considered. RCT research focuses on randomness, and the existence of ethical problems makes such research difficult. Therefore, we found few relevant RCT studies, which led to higher heterogeneity. 

Then, the search was not comprehensive enough. This was due to the imperfect retrieval strategy: only limited network databases (Cochrane Library, PubMed, Web of Science) were used in this meta‐analysis without searching published physical books.

In conclusion, although each therapy tends to have relatively better performance in some of the above aspects, it is difficult to judge which of the two is superior: there was no statistically significant difference between the two treatments. The current limitations of this meta‐analysis, mentioned above, indicate that it is necessary to increase the sample size and improve the retrieval strategy to reduce heterogeneity and bias. Further studies are necessary to assess and compare the efficiency and safety of TAR and AAD.
